# Adsorption of Zinc Ions from Aqueous Solutions on Polymeric Sorbents Based on Acrylonitrile-Divinylbenzene Networks Bearing Aminophosphonate Groups

**DOI:** 10.3390/molecules30244805

**Published:** 2025-12-17

**Authors:** Lavinia Lupa, Aurelia Visa, Adriana Popa, Maria Valentina Dinu, Ionela Fringu, Ecaterina Stela Dragan

**Affiliations:** 1 Faculty of Chemical Engineering, Biotehnologies and Environmental Protection, Politehnica University Timisoara, 6 Vasile Parvan Blvd., 300223 Timisoara, Romania; lavinia.lupa@upt.ro; 2 “Coriolan Drăgulescu” Institute of Chemistry, 24 Mihai Viteazu Blvd., 300223 Timisoara, Romania or avisa@acad-icht.tm.edu.ro (A.V.); mcreanga@acad-icht.tm.edu.ro (I.F.); 3 Petru Poni Institute of Macromolecular Chemistry, 41A Aleea Grigore Ghica Voda, 700487 Iasi, Romania; vdinu@icmpp.ro

**Keywords:** acrylonitrile-divinylbenzene networks, aminophosphonate, polymeric adsorbents, adsorption, zinc ions

## Abstract

Contamination of natural water sources with zinc ions poses serious ecological and health risks due to its toxicity and persistence. In this context, this study presents the preparation of new adsorbents based on acrylonitrile-divinylbenzene networks functionalized with aminophosphonate groups, selected for their strong chelating affinity towards Zn(II) ions. Both unmodified and functionalized materials were evaluated in adsorption experiments towards zinc ions. The adsorption capacity was evaluated as a function of the contact time and the initial zinc concentration. The functionalized adsorbents exhibited a significantly higher adsorption of zinc, attributed to the presence of aminophosphonate groups. In case of functionalisation with ethyl phosphonate group is achieved a maximum adsorption capacity of 101 mg/g. The equilibrium data followed the Langmuir isotherm, indicating monolayer adsorption, while the kinetic analysis followed the pseudo-second-order model, consistent with chemisorption. The optimal contact time was 60 min. Functionalized polymeric supports show strong potential for zinc ion removal, supporting their use in environmental remediation. Overall, the results demonstrate that aminophosphonate-functionalized polymers are highly effective adsorbents for the removal of zinc ions from contaminated waters.

## 1. Introduction

The worldwide shortage of water resources, exacerbated by pollution from industrial, agricultural and human activities, represents a serious and growing environmental challenge [1]. Factors such as population growth, uncontrolled urbanization and the excessive use of chemicals further contribute to the degradation of water quality [2]. In this context, the development of efficient technologies for the elimination of aquatic pollutants is crucial. Among these pollutants, heavy metal ions are of particular concern due to their high toxicity, persistence and bioaccumulation capacity [3]. Heavy metal ions can cause serious pathologies, highlight the need and importance of monitoring and implement effective remediation strategies to both protect public health and water resources. The World Health Organization (WHO) and the Environmental Protection Agency (EPA) have established certain permissible limits for the discharge of heavy metal ions into natural waters [4,5]. Zinc, although an essential element for the body involved in the synthesis of enzymes, bone and tissue growth, and numerous biochemical processes, can become harmful when present in excess. The main industrial sources of zinc are: galvanizing, paper and pulp industry, steel production, metallurgy, the chemical industry, and the production of paints and fertilizers [6]. High exposure to zinc can cause health problems such as headaches, nausea, skin irritations, fever, and anemia. The WHO recommends a maximum permissible limit of 5.0 mg/L of zinc in wastewater [4].

There are multiple processes for the removal of contaminants: coagulation/flocculation, precipitation, ion exchange, adsorption, etc. [7,8]. Special attention is paid to adsorption, which is an efficient and cost-effective method for removing heavy metal ions from polluted waters, generating high-quality effluents [2]. A major advantage of adsorption is the possibility of regenerating and reusing adsorbents, which reduces both operational costs and environmental impact, an especially important factor for countries with limited resources [5,9,10]. Unconventional adsorbents, such as agricultural waste and industrial sludge, are more accessible and efficient due to their favourable chemical properties. Biochar, another promising material that is obtained from carbonized biomass, has a porous structure, a large surface area and a low cost [2,11]. However, these adsorbents have limited regeneration capacity. The development of efficient regeneration methods could enhance both economic and environmental sustainability, the adsorbent reuse, and the recovery of metal ions [1]. Recent advances in the field of polymers have led to the development of synthetic polymeric adsorbents [12,13], which are characterized by tunable porosity, large surface area, dimensional stability, and fast adsorption kinetics. These adsorbents present a multitude of advantages, such as: availability in various forms (beads, membranes, fibers), the possibility of surface modification by methods such as functionalization, as well as high selectivity, obtained by attaching specific ligands that allow targeted adsorption of certain compounds [14]. Macrocross-linked materials based on styrene-divinylbenzene copolymers (S-DVB) were obtained by copolymerization of S and DVB in the presence of organic diluents, resulting in copolymers with a large surface area and high porosity [15,16,17]. Functionalization of these copolymers with aminophosphonate groups has yielded a new class of functionalized macroreticular supports with improved properties and increased selectivity towards certain organic molecules or bacteria [18,19].

Multicomponent reactions (MCRs) are processes that involve three reactants that combine in a single step to form complex products with minimal by-products. These reactions have attracted significant attention in the synthesis of functional polymers [20]. Thus, the Kabachnik-Fields (KF) reaction, first reported by M. I. Kabachnik and E. K. Fields in 1952 [20], can be considered a multicomponent reaction. In this reaction, an aldehyde, an amine, and a phosphite participate to produce an α-aminophosphonate. The α-aminophosphonate structure obtained through the KF reaction exhibits a wide range of biological activities and has also demonstrated promising metal-chelating properties, making it valuable for both biomedical and materials science applications [20].

In a previous study, we reported both the synthesis of porous anion exchangers bearing primary amine moieties based on acrylonitrile-15%divinylbenzene (AN-15%DVB) copolymers and their functionalization with aminophosphonate groups through the KF method, obtaining materials with antibacterial activity against bacterial cultures of *E. coli* and *S. aureus* [21]. These materials showed a significant antibacterial effect for reducing environmental impact. Based on our previous research [21], this article presents the functionalization with aminophosphonate groups of a weak anion exchanger derived from a gel like copolymer AN-10%DVB, having the code SV, by a KF reaction obtaining novel polymer chelators and the application of these materials in the adsorption of Zn^2+^ ions. The primary goal of the present research is to enhance the adsorption capacity and efficiency of a weak anion exchanger derived from the AN-10%DVB copolymer by functionalizing it with aminophosphonate groups through the Kabachnik-Fields (KF) reaction. This study specifically aims to develop novel polymer chelators that exhibit improved binding affinity for Zn^2+^ ions, thereby addressing the critical issue of heavy metal ion contamination in aqueous environments. Thus, this research enhances the current understanding of how heavy-metal ions interact with functionalized polymeric materials and also establishes a foundation for developing efficient and sustainable strategies for the remediation of contaminated water resources. Ultimately, the goal is to provide insights that can guide future studies aimed at optimizing polymeric support for the removal of various contaminants, thereby contributing to environmental protection and public health.

## 2. Results and Discussion

### 2.1. Chemical and Morphological Characterization of Polymer Chelators

**Scheme 1:**
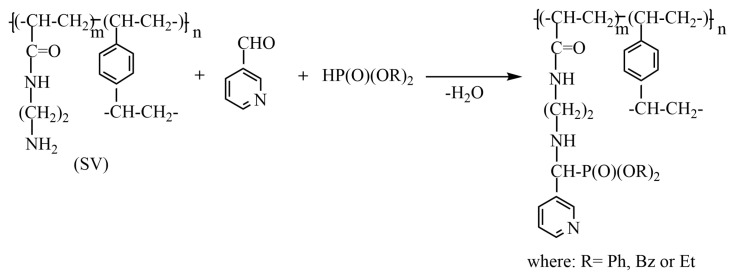
Reaction of the final products, aminodibenzylphosphonate groups (BzSV), aminodiethylphosphonate groups (EtSV) and aminodiphenylphosphonate groups (PhSV).

The chemical structures of the functionalized copolymers: aminodibenzylphosphonate groups (BzSV), aminodiethylphosphonate groups (EtSV) and aminodiphenylphosphonate groups (PhSV), are illustrated in Scheme 1. These materials are based on AN-10%DVB copolymers that have been chemically functionalized with aminophosphonate groups. The incorporation of aminophosphonate groups confers chelating properties, increasing the affinity of the copolymers for divalent metal ions, such as zinc ions.

**Figure 1:**
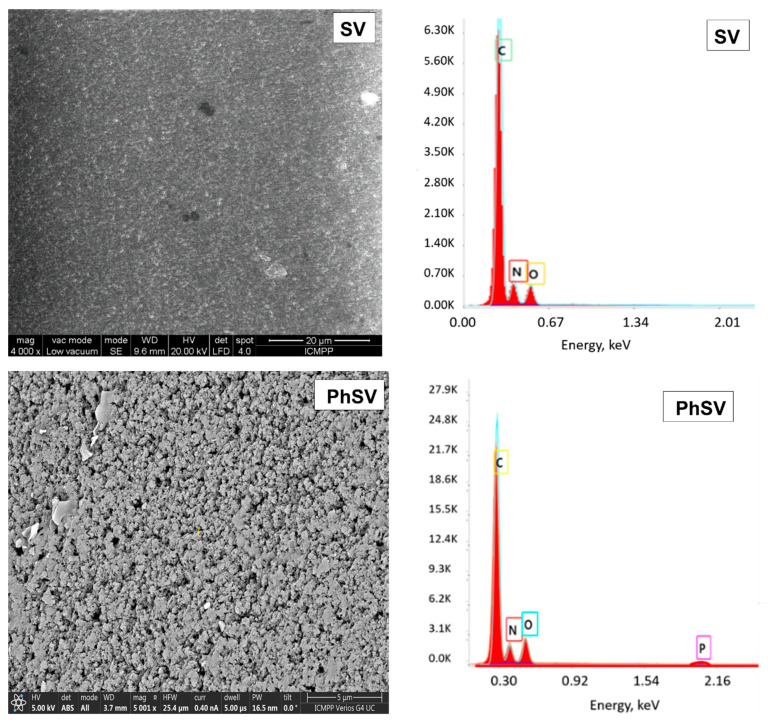

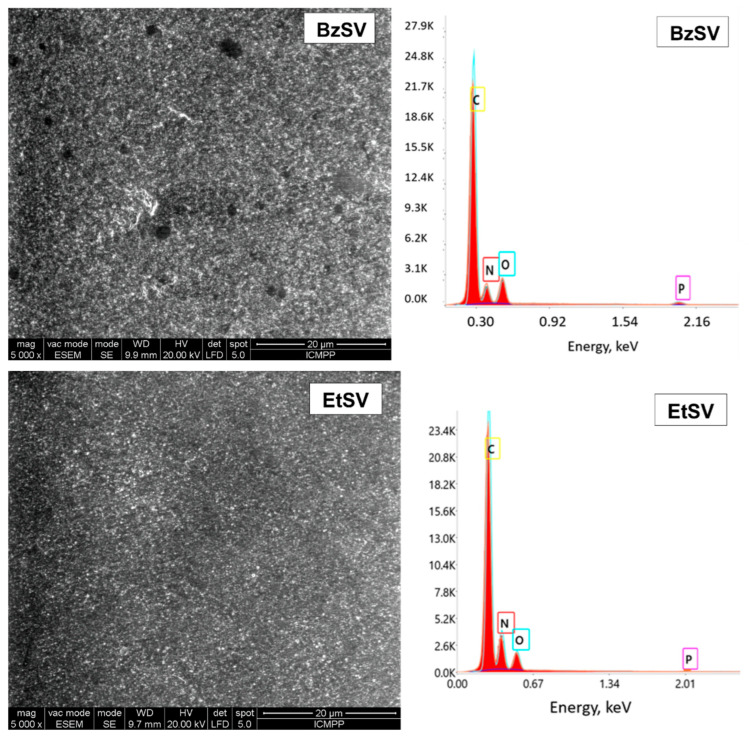
SEM images and EDX spectra of the networks before and after functionalization; mag. 5000×; scaling bare 200 μm.

SEM and EDX images of the SV anion exchanger, both before and after the functionalization process, are shown in Figure 1. From the SEM images, it is obvious that the pristine network display a homogeneous surface. However, a certain roughness was observed after the functionalization through the KF reaction, which suggests the generation of heterogeneity in their morphology after functionalization. This roughness of the copolymers leads to a more efficient interaction between the adsorbent materials and zinc ions, and higher adsorption capacities were obtained with the functionalized copolymers compared to the pristine copolymer, as it will be shown below. The EDX spectra (Figure 1) indicate that the copolymer has been functionalized with aminophosphonate groups. The elemental analysis values obtained from the semi-quantitative EDX determination are presented in Table 1. The degree of functionalization for each copolymer sample was determined from the semi-quantitative EDX analysis. The functional group content for the obtained products was: 11.5 mmol/g for PhSV, 12.6 mmol/g for BzSV, and 14.0 mmol/g for EtSV.

From the semi-quantitative analysis, an increase in carbon content, the appearance of phosphorus, and a slight decrease in nitrogen and oxygen content are observed.

**Figure 2:**
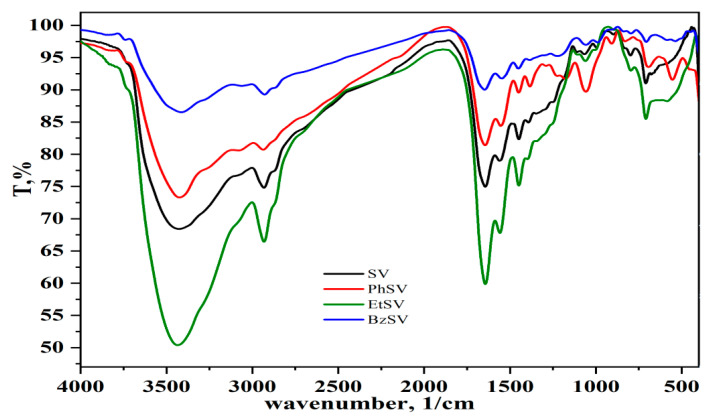
FTIR spectra of polymer networks before and after functionalization with aminophosphonate groups.

The FTIR spectra of the pristine anion exchanger (SV) and of the functionalized copolymers are presented in Figure 2.

The FTIR spectrum of the pristine SV anion exchanger showed the following bands: 1449 cm^−1^ and 710 cm^−1^ and were assigned to N-H groups, while the bands located at 3429 cm^−1^ and 1643 cm^−1^ were assigned to -NH_2_ groups, and C=O bond in the secondary amide bond in SV copolymer (amide I). The FTIR spectrum of the BzSV sample showed adsorption bands in the following regions: 3510–3350 cm^−1^ assigned to groups (aliphatic–NH and -OH), 1680–1570 cm^−1^ assigned to groups (-C=C, aromatic ring, C=O in the secondary amide bond, and the bending vibration of N-H bonds in amine groups), 1360–1210 cm^−1^ assigned to groups (γN-aliphatic, γP=O bond), 1169–950 cm^−1^ assigned to groups (γP=O) and 880–709 cm^−1^ assigned to groups (N-H groups in primary amine and P-OR). For the EtSV sample, significant adsorption bands were observed in the following regions: 3440–3320 cm^−1^ assigned (to aliphatic –OH and NH groups), 1650–1555 cm^−1^ assigned (C=O in the secondary amide bond and the bending vibration of N-H bonds in amine groups), 1458–1370 cm^−1^ assigned (γP=O) and 908–680 cm^−1^ assigned (to N-H groups in primary amine and P-OR). The FTIR spectrum of the PhSV sample showed adsorption bands in the following regions: 3545–3350 cm^−1^ assigned to (aliphatic -OH and NH groups), 1660–1569 cm^−1^ assigned to (C=O in the secondary amide bond and the bending vibration of N-H bonds in amine groups), 1460–1390 cm^−1^ assigned to (γP=O) and 880–690 cm^−1^ assigned to N-H groups in primary amine and P-OR [18,19,21,22].

**Figure 3:**
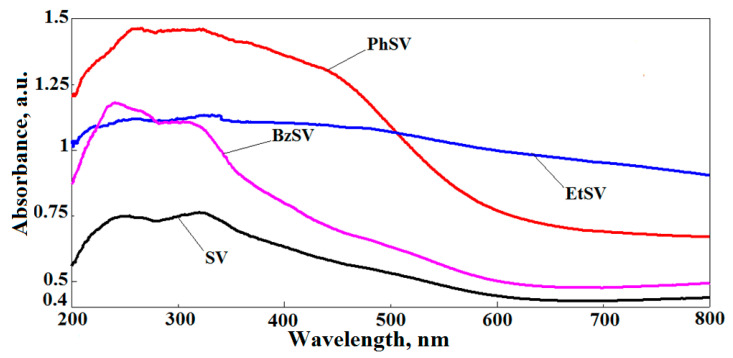
UV–Vis Spectra of pristine SV and functionalized derivatives (BzSV, EtSV, PhSV).

UV–Vis spectra of the SV anion exchanger, before and after functionalization with aminophosphonate groups (BzSV, EtSV, and PhSV), are presented in Figure 3.

A weak absorption band observed for the pristine SV anion exchanger, located in the range of 250–350 nm, is most likely attributed to n–π* transitions originating from lone pairs on the amino groups [23]. All copolymers resulting from functionalization with aminophosphonate groups (BzSV, EtSV and PhSV), exhibit broad and intense absorption bands in the high-energy region (200–450 nm). These absorption features are mainly associated with π–π* transitions of the aromatic rings [23]. In addition, contributions from n–π* transitions originating from lone pairs on the aminophosphonate groups are also evident, suggesting functionalization with aminophosphonate groups.

**Figure 4:**
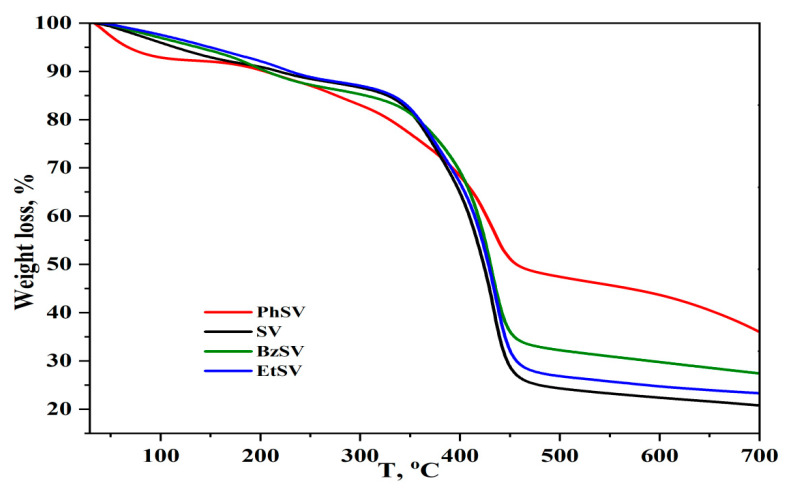
TG curves for SV and functionalized networks (EtSV, BzSV and PhSV) recorded in nitrogen atmosphere.

The thermal analysis data obtained for the pristine SV anion exchanger and functionalized copolymers with the aminophosphonate group (PhSV, EtSV, BzSV), up to 700 °C, is presented in Figure 4.

For the SV anion exchanger, a percentage of 11.74% residue was found at 700 °C after a total mass loss of 88.26%. The highest thermal stability was found for PhSV, with the highest residue (36%) and the lowest mass loss (64%). EtSV and BzSV show moderate stability, BzSV (lost 72.59% of its total mass, leaving 27.41% residue) being less thermally stable than EtSV (with a residue of 23.30% and a total mass loss of 76.70%). Functionalization of AN-DVB copolymer with aminophosphonate groups improves the thermal stability of the SV pristine copolymer [21].

### 2.2. Adsorption Study

#### 2.2.1. Kinetic Studies

**Figure 5:**
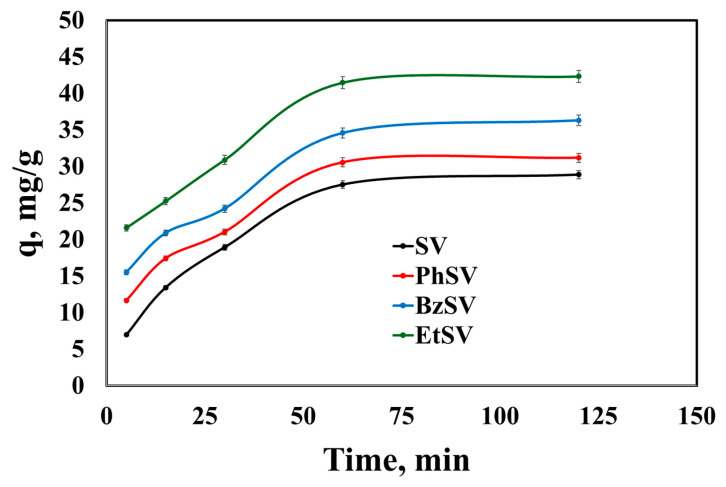
Variation in adsorption capacity as a function of contact time for polymer networks tested in the removal of zinc ions from aqueous solution. Sorption conditions: T = 25 °C, adsorbent dose 1 g/L; C_Zn_ = 50 mg/L, pH = 6.

To assess the equilibrium time between the adsorbent and zinc ions, the impact of stirring time on the adsorption performance of the studied polymers support in the removal of Zn^2+^ ions from aqueous solutions was investigated. The findings are illustrated in Figure 5.

It can be observed that initially, the adsorption capacity exhibits a linear increase with the extension of contact time. However, after 60 min, the rate of increase in adsorption capacity becomes gradual, ultimately reaching a plateau. This indicates that equilibrium between the polymers support and zinc ions is achieved at 60 min, which was therefore selected as the optimal reaction time for subsequent experiments.

The experimental data collected at various time intervals can be utilized for the design and modelling the adsorption process. The most commonly employed kinetic models for this purpose are the pseudo-first order (PFO) and pseudo-second order (PSO) kinetic models. The linear forms of these kinetic models are represented by the following equations [24,25]:

(1) $$\ln\left(q_{e}-q_{t}\right)=lnq_{t}-k_{1}t$$

(2) $$\frac{t}{q_{t}}=\frac{1}{k_{2}q_{e}^{2}}+\frac{t}{q_{t}}$$

where q_t_ and q_e_ represent the amount of Zn^2+^ adsorbed on the polymer supports at the time t and at equilibrium, respectively, mg/g; k_1_ represents the PFO adsorption rate constant, min^−1^; k_2_ represents the PSO adsorption rate constant, min^−1^ (mg/g)^−1^.

**Figure 6:**
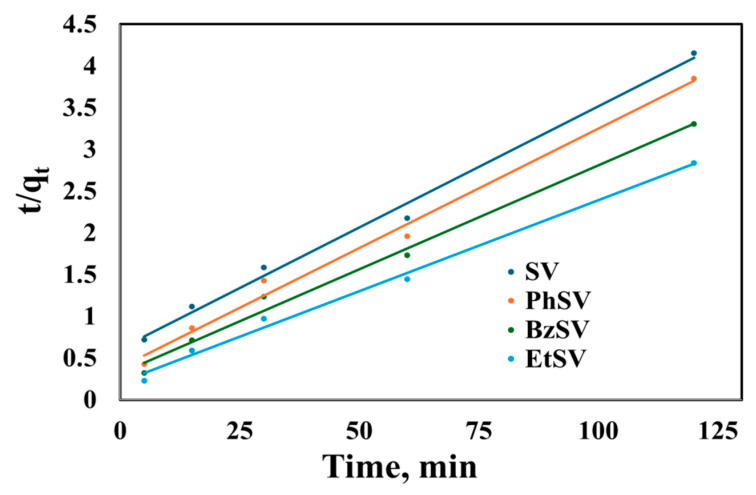
PFO kinetic plot for Zn^2+^ adsorption onto polymer networks.

**Figure 7:**
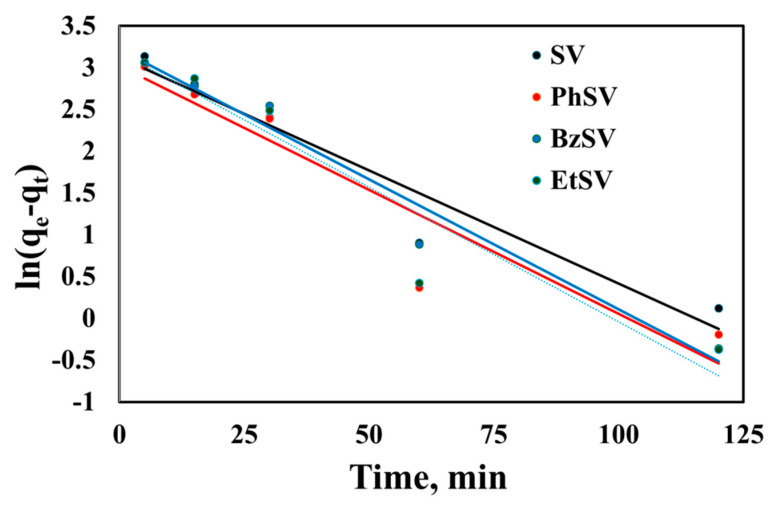
PSO kinetic plot for Zn^2+^ adsorption onto polymer networks.

The PFO rate constant (k_1_) and the equilibrium adsorption capacity (q_e_) can be determined from the slope and intercept of the graphical representation of ln(q_e_ − q_t_) vs. time (t), as shown in Figure 6. Additionally, the PSO rate constant (k_2_) and the equilibrium adsorption capacity (q_e_) were derived from the plot of t/q_t_ versus time (t), illustrated in Figure 7. The values of the rate constants, along with the corresponding regression coefficients (R^2^) obtained for both models, are summarized in Table 2.

The data presented in Table 2 indicate that the kinetics of Zn^2+^ removal via adsorption onto the studied polymer supports is best described by a PSO kinetic model. This conclusion is supported by the correlation coefficient, which is very close to 1, indicating a strong linear relationship between the variables involved. Furthermore, the theoretically predicted equilibrium adsorption capacity (q_e_) closely aligns with the experimentally determined value, reinforcing the validity of the PSO model.

The PSO kinetic model suggests that the rate of adsorption is dependent on the square of the concentration of the adsorbate (Zn^2+^ ions) at the interface. This implies that the adsorption process is likely governed by chemical interactions rather than purely physical adsorption. In this context, the rate-determining step may involve chemisorption, characterized by the formation of chemical bonds between the adsorbent (functional groups of the polymer support) and the adsorbate (Zn^2+^ ions). Such interactions often involve valence forces, which may occur through the sharing or exchange of electrons between the zinc ions, and the functional groups present on the polymer surface [26,27,28]. This mechanism highlights the significance of chemical affinity in the adsorption process, suggesting that the polymer supports possess specific binding sites that facilitate the effective removal of Zn^2+^ ions from aqueous solutions.

#### 2.2.2. Equilibrium Studies

**Figure 8:**
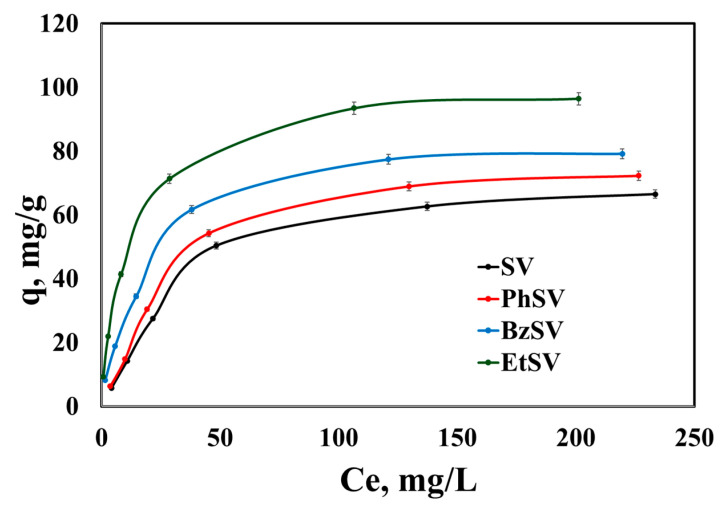
Equilibrium isotherms of in the adsorption process of zinc from aqueous solutions onto the studied polymer networks. Sorption conditions: T = 25 °C, adsorbent dose 1 g/L, C_Zn_ = 10–300 mg/L, t = 60 min, pH = 6.

The equilibrium data obtained from experiments and presented in Figure 8 highlight the variation in the adsorption capacity of functionalized polymer supports as a function of the equilibrium concentration of Zn^2+^ in aqueous solutions, with the adsorption capacity increasing as the concentration of Zn^2+^ ions rises. This phenomenon occurs because a higher concentration of Zn^2+^ ions leads to greater availability of adsorbate, enhancing the likelihood of adsorption events on the polymer supports. Additionally, the increased concentration creates a stronger concentration gradient that drives more Zn^2+^ ions towards the polymer surface. Initially, this results in the effective utilization of active sites on the functional polymer, and at higher concentrations, multi-layer adsorption may occur, further boosting capacity.

Furthermore, stronger ionic or coordination bonds can form at elevated concentrations, enhancing the retention of zinc ions. However, for concentrations greater than 75 mg/L, a constant level of adsorption is reached, indicating saturation of the adsorption sites.

To characterize the equilibrium established between the liquid phase and the polymeric adsorbent, as well as to determine the maximum adsorption capacity, the experimental results were analyzed using the Langmuir and Freundlich isotherm models in their linear forms [24,25].

(3) $$\frac{C_{e}}{q_{e}}=\frac{1}{k_{L}q_{m}}+\frac{C_{e}}{q_{m}}$$

(4) $$\ln q_{e}=lnK_{F}+\frac{1}{n}lnC_{e}$$

where *q_e_* is the adsorption capacity at equilibrium and *q_m_* is the maximum adsorption capacity of polymer support in the removal process of Zn^2+^ ions from aqueous solutions (mg/g); *C_e_* is Zn^2+^ equilibrium concentration (mg/L); *K_L_* Langmuir constant associated with energy of adsorption (L/mg); *K_F_* is the Freundlich constant (mg/g); 1/n represents dimensionless heterogeneity factor.

The Langmuir isotherm model is based on the assumption of a monolayer adsorption on a surface with a finite number of identical sites, suggesting that once a site is occupied by an adsorbate molecule, no further adsorption can occur at that site. This model is characterized by a maximum adsorption capacity, which represents the saturation point of the adsorbent. By applying these models to the experimental data, we can derive key parameters such as the maximum adsorption capacity (*q_m_*) and the affinity constant for the adsorption process. The comparative analysis of these models provides insights into the nature of the adsorption process and the suitability of the polymeric adsorbent for removing contaminants, such as Zn^2+^ ions, from aqueous solutions. In contrast, the Freundlich isotherm model, which is applicable for heterogeneous surfaces, assumes that the adsorption capacity increases with the concentration of the adsorbate in a non-linear manner, reflecting the multilayer adsorption process. This model is particularly useful in describing systems where the energy of adsorption varies across the surface of the adsorbent.

**Figure 9:**
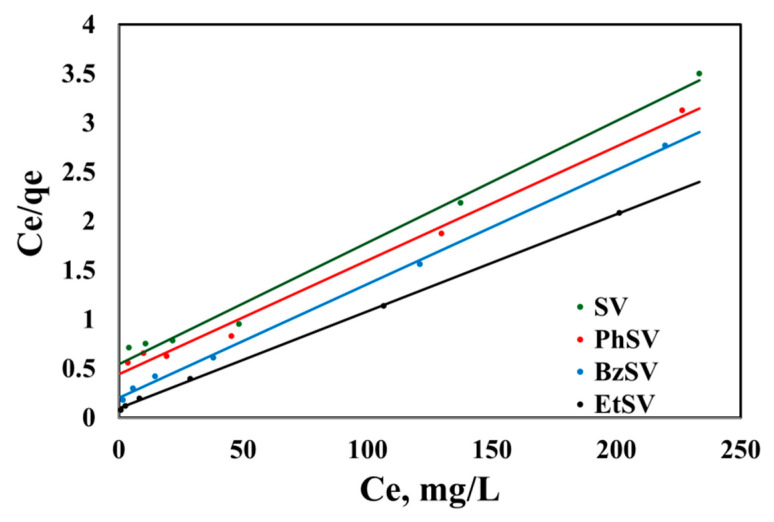
Langmuir isotherm for Zn^2+^ adsorption onto polymer networks.

**Figure 10:**
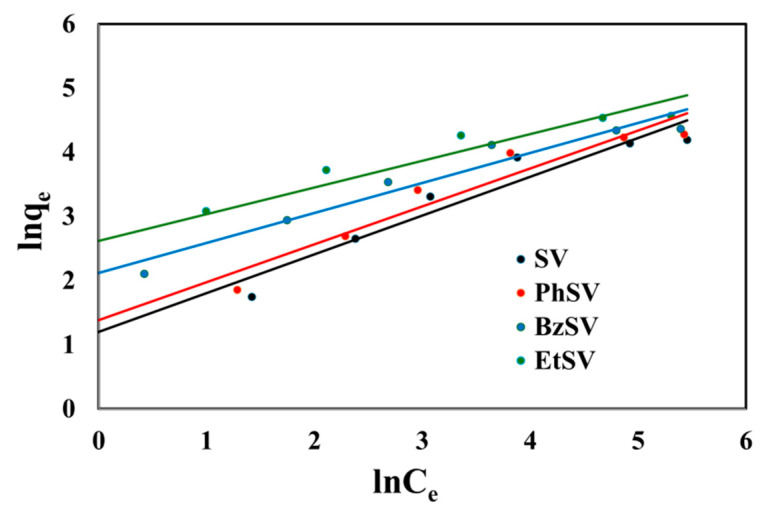
Freundlich isotherm for Zn^2+^ adsorption onto polymer networks.

The isotherms, plotted according to their respective linearized equations, are illustrated in Figure 9 and Figure 10. The values of the isotherm parameters derived from these plots, along with their corresponding correlation coefficients, are summarized in Table 3. By plotting C_e_/q_e_ vs. C_e_, the slope and intercept of the resulting linear plot allow the calculation of q_m_ and K_L_ (Figure 9). By plotting ln q_e_ against ln C_e_ (Figure 10), the slope and intercept of the resulting linear graph provide the values for K_F_ and 1/n.

The Langmuir model has proven to be suitable for describing the adsorption process of Zn^2+^ ions onto the analyzed materials, as evidenced by the correlation coefficients obtained, which are very close to unity in all cases. This indicates a strong correspondence between the experimental data and the theoretical model across the entire range of tested concentrations. Moreover, the estimated maximum adsorption capacities derived from the Langmuir plots align well with the values determined experimentally. This close agreement suggests a uniform distribution of the adsorbed species on the surface of the materials, which can be associated with a homogeneous distribution of active adsorption sites. The absence of a trend indicating migration of the adsorbed species on the solid surface also supports the possibility of a chemisorption mechanism, wherein the interactions between the polymeric support and the adsorbed component, Zn^2+^, are of a chemical nature rather than purely physical. Furthermore, the Langmuir model demonstrates a better fit compared to the Freundlich model for the adsorption of Zn^2+^ ions onto the studied polymeric supports. Notably, the Freundlich constant 1/n is less than one for all the examined polymeric supports, indicating favourable adsorption conditions. This suggests that while the Freundlich model captures some aspects of the adsorption process, the Langmuir model more accurately reflects the underlying mechanisms of adsorption, particularly in terms of the uniformity of active sites and the nature of the interactions involved.

#### 2.2.3. Influence of the Nature of Polymeric Supports on Their Adsorptive Properties

**Figure 11:**
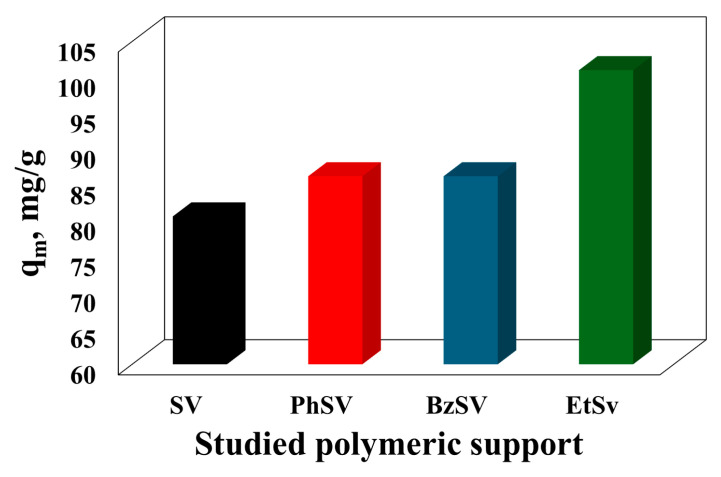
Dependence of the adsorptive properties of the studied polymer networks on their structure.

To identify the most effective polymeric support for the removal of Zn^2+^ ions from aqueous solutions, the maximum adsorption capacities, determined from the Langmuir isotherm for all adsorbents, were compared in Figure 11.

As can be observed in Figure 11, the functionalization with aminophosphonate groups of the AN-DVB (10%) polymeric networks, which initially possess primary amine groups, enhances the adsorption capacity of the resulting adsorbent material. Among the utilized phosphonate groups, the ethyl phosphonate group proved to be the most effective, probably due to its smaller size and compact shape, which allow it to approach the surface of the polymeric support and Zn^2+^ ions more efficiently. The steric and electronic interactions of the benzyl and phenyl aromatic groups can hinder the mobility and overall efficiency of zinc adsorption. These larger groups may create steric hindrance, preventing optimal contact between the adsorbent and the Zn^2+^ ions. In contrast, the ethyl phosphonic group provides favourable polarity, which facilitates more effective interactions with the Zn^2+^ ions. This increased polarity enhances the electrostatic and chemical interactions between the adsorbent and the target ions, leading to improved adsorption performance [26,27]. Overall, the choice of functional groups significantly impacts the adsorption characteristics of the polymeric supports. The findings suggest that optimizing the functionalization of polymeric materials can lead to more effective adsorbents for the removal of specific contaminants, such as Zn^2+^ ions, from aqueous solutions.

**Figure 12:**
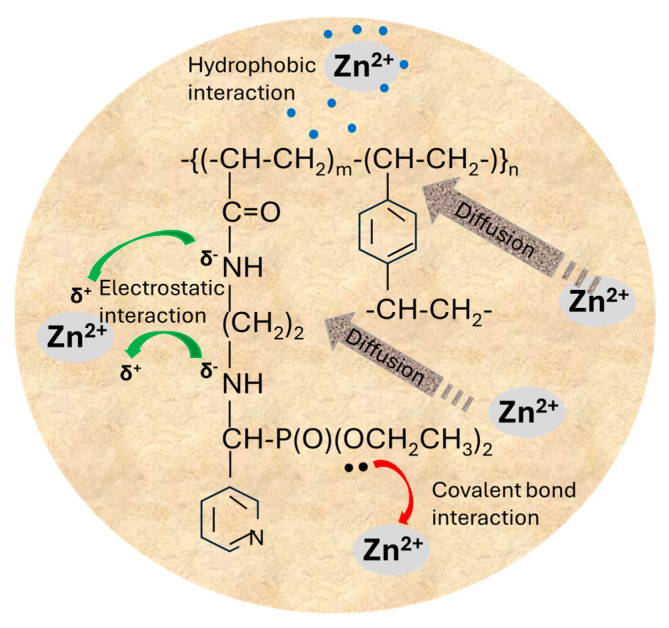
Sorption mechanism of zinc ions onto EtSV.

The sorption mechanism of zinc ions onto AN-DVB (10%) polymeric networks functionalized with ethyl phosphonate groups is illustrated in Figure 12. The acrylonitrile-divinylbenzene (AN-DVB) networks functionalized with ethyl phosphonate groups exhibit several potential interactions with zinc ions that facilitate adsorption. Firstly, the ethyl phosphonate groups provide active sites for coordination through the lone pairs on the oxygen atoms, forming coordinate covalent bonds with zinc ions. Additionally, electrostatic interactions occur due to the positively charged zinc ions attracting negatively charged sites within the polymer matrix, enhancing the binding affinity. Hydrophobic interactions may also play a role, as the polymer’s hydrophobic regions can promote the displacement of water molecules surrounding the zinc ions, favoring their attachment to the polymer surface. Furthermore, the diffusion of zinc ions through the polymer network to reach these active sites is influenced by concentration gradients and the physical properties of the polymer. Collectively, these interactions contribute to the effective adsorption of zinc ions onto the functionalized AN-DVB networks.

Utilizing the adsorbent material in multiple cycles of adsorption and desorption is crucial for evaluating its efficiency and sustainability in practical applications. The ability to regenerate the material and maintain its performance over several cycles is indicative of its long-term viability as an effective adsorbent for zinc removal. The adsorbent material, EtSV, was regenerated through treatment with 1 M HNO_3_, which demonstrated the best performance for desorption of zinc ions. This regenerated material was subsequently utilized in three cycles of adsorption and desorption. The adsorption capacity remained consistent during the first two cycles, indicating effective regeneration and stability of the adsorbent material. However, in the third cycle, a decrease in adsorption capacity of approximately 7% was observed. This reduction may be attributed to potential changes in the surface characteristics of the adsorbent or the cumulative effects of previous adsorption cycles. Overall, these results suggest that while the EtSV material exhibits good reusability, some decline in performance can occur after multiple cycles of use.

The adsorption capacity of the AN-DVB (10%) polymeric networks, functionalized with ethyl phosphonate groups, was evaluated against the maximum adsorption capacity derived from the Langmuir isotherm for other materials discussed in the literature concerning the adsorption of zinc from aqueous solutions. The results are presented in Table 4. It was observed that the studied material exhibited significantly superior adsorption capacity.

This leads to the conclusion that the AN-DVB (10%) polymeric networks functionalized with ethyl phosphonate groups are highly effective for the removal of zinc ions from aqueous solutions. The enhanced adsorption performance can be attributed to the specific interactions between the ethyl phosphonate functional groups and zinc ions, which facilitate a more efficient binding process compared to other materials previously studied. This finding suggests that the functionalization of polymeric networks can be a promising strategy for improving the adsorption characteristics of materials used in environmental remediation applications.

## 3. Materials and Methods

### 3.1. Materials

Divinylbenzene (DVB), technical grade—comprising 64% ortho-, meta-, and para-DVB isomers and 36% ethylstyrene as determined by gas chromatography—was purchased from Purolite (Victoria, Romania) and used as received. Benzoyl peroxide (BPO), obtained from Fluka (Buchs, Switzerland), served as the radical polymerization initiator after being recrystallized twice from methanol. Acrylonitrile (AN), acquired from Sigma-Aldrich (Burlington, MA, USA), was distilled at 76–77 °C under atmospheric pressure (101.3 × 10^3^ Pa). 2-Ethyl-1-hexanol (2-EH) technical grade, was distilled at 183.5 °C and 101.3 × 10^3^ Pa. 1,2-Diaminoethane (EDA), also from Sigma-Aldrich, was distilled at 118 °C prior to use. Dimethylformamide (DMF, Sigma-Aldrich) was used without further purification. Hydrochloric acid (HCl) and sodium hydroxide (NaOH) were supplied by Chemical Company (Iași, Romania) and used as received. Diphenylphosphite (Aldrich, technical grade ≥ 85%), 3-pyridinecarboxaldehyde (Aldrich, 98%), anhydrous tetrahydrofuran (THF, Sigma-Aldrich, >99.9%), and ethanol p.a. (Chimopar, Bucharest, Romania) were employed as reagents in the synthesis procedures.

### 3.2. Equipments

Fourier-transform infrared (FTIR) spectroscopy was performed using KBr pellet samples on a JASCO FTIR spectrophotometer (JASCO, Tokyo, Japan) over the range of 4000–400 cm^−1^. The Ultraviolet-visible (UV-vis) spectra of solid samples (previously ground using a mortar and pestle) were recorded at room temperature using a JASCO V-650 spectrometer (JASCO GmbH, Schwentinental, Germany), equipped with an integrating sphere that allows the collection of diffuse reflectance. Calibration was performed using PTFE (polytetrafluoroethylene), commonly known as Spectralon, a highly reflective, stable white material commonly used as a reference standard in UV-VIS diffuse reflectance measurements. Thermogravimetric analysis (TGA) was conducted on a TGA/SDTA 851-LF1100 instrument (Mettler Toledo, Greifensee, Switzerland) from 25 to 700 °C at a heating rate of 10 °C/min under a nitrogen atmosphere. The elemental composition of the copolymers was determined by energy-dispersive X-ray (EDX) analysis using an Octane Elect Super SDD detector (Ametek, Berwyn, PA, USA) mounted on a Verios G4 UC scanning electron microscope (Thermo Scientific, Brno, Czech Republic).

### 3.3. Preparation of Pure SV Copolymer with Primary Amine Groups

First, the AN-10%DVB copolymer was synthesized using 2-EH as diluent agent, following a previously established protocol [21]. The synthesis was carried out by suspension polymerization, starting with 2 h at 60 °C, followed by 3 h at 70 °C, and then 5 h at 85 °C. The resulting copolymer beads were washed thoroughly with water. The diluent and PAN homopolymer were removed via extraction first with methanol and then with DMF. Finally, the selected beads (0.3–1.0 mm) were washed with methanol and dried at room temperature for 24 h, and 48 h under vacuum at 50 °C. The anion exchanger containing primary amine groups (code SV) was prepared via the aminolysis–hydrolysis reaction of the nitrile groups with EDA, conducted over 10 h at 118–120 °C, using a molar ratio CN:EDA:H_2_O of 1:10:2. Finally, the beads were filtered and washed with distilled water up to the neutral pH. The anion exchange capacity was evaluated according to the previously presented protocol and was about 2.93 milliequivalents per gram (meq g^−1^) [21].

### 3.4. Functionalization of Amino Groups in AN-DVB Copolymers with Aminophosphonate Groups

In this work, we used a previously described procedure [21] to introduce aminophosphonate groups onto the anion exchanger bearing primary amine groups (SV) derived from the AN-10%DVB copolymer was functionalized by a one-step Kabachnik-Fields reaction using diphenylphosphite and pyridinecarboxaldehyde, obtaining functionalized copolymer with aminodiphenylphosphonate groups (PhSV). The reaction was carried out in tetrahydrofuran at 60 °C, using a 1:1.3:1.3 molar ratio for polymer:aldehyde:phosphite. After 40 h, the reaction mixture was filtered, washed with ethanol and dried at 50 °C for 24 h. The functionalized copolymer with aminodibenzylphosphonate groups (BzSV) and the functionalized copolymer with aminodiethylphosphonate groups (EtSV) were obtained by the previously reported procedure [21].

### 3.5. Adsorption of Zinc Ions

The research on the adsorption of Zn^2+^ ions from aqueous solutions on the synthesized materials was carried out in batch mode. A specified amount of adsorbent (0.025 g) was added to 25 mL of the zinc solution, which was contained in 50 mL glass Erlenmeyer flasks. The mixture was then agitated to ensure thorough contact between the adsorbent and the zinc ions, allowing for efficient sorption to occur, at a constant speed of 200 rpm (this speed being maintained in all experiments), using a Julabo SW23 stirring bath (Seelbach, Germany), at 25 °C. The adsorbent properties of the investigated materials were evaluated depending on the initial zinc concentration, the contact time between the adsorbent and the adsorbate, as well as the nature of the adsorbent material. The adsorption capacity of the studied materials was calculated using Equation (5):

(5) $$q_{t}=\frac{\left(C_{0}-C_{t}\right)\cdot V}{m}$$

where *C*_0_ and *C_t_* are the zinc concentrations (mg/L) in the initial solution (t = 0) and after a certain contact time *t, V* is the volume of the solution (L), and *m* is the mass of the adsorbent (g). To evaluate the influence of zinc concentration on the adsorptive capacity of the investigated materials, solutions with zinc concentrations varying in the range (10–300 mg/L) were used, keeping constant the other parameters (pH = 6, solid:liquid ratio = 1:1, stirring time of 1 h). The solutions with known concentrations of zinc were prepared from a stock solution of 1 g/L zinc nitrate. This stock solution served as the basis for diluting to the desired concentrations for the adsorption experiments. It was chose to conduct the experiments at a neutral pH (pH = 6) because it is well-established that at acidic pH, competition occurs between Zn^2+^ ions and H^+^ ions. This competitive interaction can significantly hinder zinc adsorption. Conversely, at higher alkaline pH levels, zinc ions tend to precipitate, particularly in more concentrated solutions, leading to removal through precipitation rather than adsorption [32,34]. This approach not only optimizes zinc adsorption but also facilitates the potential discharge of treated waters without the need for an additional pH adjustment step prior to discharge. The initial pH of the zinc solutions was adjusted with 0.1 N NaOH or HCl solutions. The pH of the solutions was determined using a Metler Toledo pH meter (Greifensee, Switzerland), ensuring accurate and reliable measurements throughout the experiments. To determine the equilibrium time between the adsorbent and the adsorbate, the studies were carried out at a solid:liquid ratio of 1 g/L, using a zinc solution with a concentration of 50 mg/L. The two phases were left in contact for different time intervals (5–120 min) at 298 K. After the expiration of the time, the suspensions were filtered, and the residual zinc concentration in the solution was measured by atomic absorption spectrometry, using a Varian SpectrAA 280 FS spectrometer (Victoria, Australia). For the calibration a Merk standard solution was used and the specific wavelength used for the detection of Zn(II) by Atomic Absorption Spectroscopy (AAS) is 213.9 nm. This wavelength corresponds to the resonance line of zinc, allowing for accurate measurement of its concentration in various samples.

## 4. Conclusions

The adsorption of Zn^2+^ ions onto the functionalized polymeric networks has been thoroughly investigated in this study, highlighting the significant impact of both the nature of the polymeric material and the specific functional groups employed. The results demonstrate that functionalizing an anion exchanger bearing primary amine groups (SV), derived from an AN-10%DVB copolymer, with aminophosphonate groups markedly changed the copolymer morphology from a homogeneous surface to a heterogeneous one, facilitating the access of Zn^2+^ ions to a higher number of chelating sites, thus increasing their adsorption capacity. Among the various phosphonate groups tested, the ethyl phosphonate group emerged as the most effective due to its compact structure, which allows for closer proximity to the Zn^2+^ ions and facilitates stronger interactions. The application of the Langmuir and Freundlich isotherm models provided valuable insights into the adsorption behaviour, with the Langmuir model showing a superior fit, indicating a monolayer adsorption on a surface with finite identical sites. The results of the kinetic study indicated that the pseudo-second-order kinetic model best described the adsorption kinetics of zinc ions onto the functionalized networks. This suggests that the rate of adsorption is primarily controlled by chemical interactions rather than mass transfer limitations. The presence of steric hindrance from larger aromatic groups was noted to limit the efficiency of adsorption, underscoring the importance of selecting appropriate functional groups for optimizing adsorption performance. In conclusion, this research underscores the potential of functionalized polymeric networks as effective adsorbents for the removal of Zn^2+^ ions from aqueous solutions. The findings contribute to the development of advanced materials for environmental remediation, paving the way for future studies aimed at optimizing polymeric supports for various contaminants and further investigating the kinetics of adsorption processes.

## Figures and Tables

**Table 1:** The EDX analysis of the pristine anion exchanger (SV) and of the aminophosphonate functionalized networks.

Element/ Sample	Wt.%			
	SV	PhSV	BzSV	EtSV
C	67.70	68.43	68.0	69.0
N	21.0	16.13	17.70	19.6
O	11.30	10.73	13.0	10.2
P	-	4.71	1.3	1.2

**Table 2:** Kinetic parameters for Zn^2+^ adsorption onto polymer networks.

Adsorbent Material	q_e, exp_, mg/g	PFO Kinetic Model			PSO Kinetic Model		
		q_e, calc_, mg/g	k_1_, min^−1^	R^2^	q_e_, Calc, mg/g	k_2_, min^−1^ (mg/g)^−1^	R^2^
**SV**	28.9	22.7	0.0271	0.9334	34.5	0.00136	0.9931
**PhSV**	31.2	20.4	0.0297	0.8848	34.9	0.00211	0.9907
**BzSV**	36.3	24.9	0.0311	0.9639	40.2	0.00195	0.9905
**EtSV**	42.3	24.1	0.0322	0.9095	45.9	0.00222	0.9933

**Table 3:** Equilibrium sorption isotherm parameters for Zn^2+^ adsorption onto polymer networks.

Adsorbent Material	q_m, exp_ mg/g	Langmuir Isotherm			Freundlich Isotherm		
		K_L_ L/mg	q_m, calc_ mg/g	R^2^	K_F_ mg/g	1/n	R^2^
**SV**	66.6	0.0228	80.6	0.9903	3.32	0.6053	0.9169
**PhSV**	72.3	0.0003	86.2	0.9915	3.98	0.5913	0.9170
**BzSV**	79.2	0.0572	86.2	0.9984	8.36	0.4665	0.9427
**EtSV**	96.5	0.1002	101	0.9995	13.7	0.4163	0.9408

**Table 4:** Comparison of the maximum adsorption capacity of different adsorbents for removal of zinc ions from aqueous solution.

Adsorbent.	q_m_, mg/g	T, K	t, min	pH	References
Fe_2_O_3_@SBA-15-CS-AEAPTMS	102.6	298	60	6	[29]
Na-Bentonite	9.45	299	60	6.5	[30]
Spirodela polyrhiza	28.5	303	120	6	[31]
CC	46.49	303	30	6	[32]
SBA-15-NN	52.3	298	40	5	[33]
ion-imprinted polymer	21.61	200	298	6	[34]
EtSV	101	60	298	6	This work

## Data Availability

Data are contained within the article.
